# The epinephrine-induced PGE2 reduces Na^+^/K^+^ ATPase activity in Caco-2 cells via PKC, NF-κB and NO

**DOI:** 10.1371/journal.pone.0220987

**Published:** 2019-08-08

**Authors:** Layla El Moussawi, Mohamed Chakkour, Sawsan Kreydiyyeh

**Affiliations:** Department of Biology, Faculty of Arts & Sciences, American University of Beirut, Beirut, Lebanon; Universidade Federal do Rio de Janeiro, BRAZIL

## Abstract

We showed previously an epinephrine-induced inhibition of the Na^+^/K^+^ ATPase in Caco-2 cells mediated via PGE2. This work is an attempt to further elucidate mediators downstream of PGE2 and involved in the observed inhibitory effect. The activity of the Na^+^/K^+^ ATPase was assayed by measuring the amount of inorganic phosphate liberated in presence and absence of ouabain, a specific inhibitor of the enzyme. Changes in the protein expression of the Na^+^/K^+^ ATPase were investigated by western blot analysis which revealed a significant decrease in the abundance of the ATPase in plasma membranes. Treating the cells with epinephrine or PGE2 in presence of SC19220, a blocker of EP1 receptors abolished completely the effect of the hormone and the prostaglandin while the effect was maintained unaltered in presence of antagonists to all other receptors. Treatment with calphostin C, PTIO, ODQ or KT5823, respective inhibitors of PKC, NO, soluble guanylate cyclase and PKG, abrogated completely the effect of epinephrine and PGE2, suggesting an involvement of these mediators. A significant inhibition of the ATPase was observed when cells were treated with PMA, an activator of PKC or with 8-Br-cGMP, a cell permeable cGMP analogue. PMA did reduce the protein expression of IκB, as shown by western blot analysis, and its effect on the ATPase was not manifested in presence of an inhibitor of NF-κB while that of SNAP, a nitric oxide donor, was not affected. The results infer that NF-κB is downstream PKC and upstream NO. The data support a pathway in which epinephrine induces the production of PGE2 which binds to EP1 receptors and activates PKC and NF-κB leading to NO synthesis. The latter activates soluble guanylate cyclase resulting in cGMP production and activation of PKG which through direct or indirect phosphorylation inhibits the Na^+^/K^+^ ATPase by inducing its internalization.

## Introduction

The effect of stress on gut functions and specifically on colonic transport processes has been well recognized. Stress alters the absorptive and secretory activities of the large intestine leading to abnormal water movements and fecal water elimination as a result of changes in electrolyte transport processes. Ion transport in the colon is regulated by hormones, neurotransmitters and inflammatory mediators, and is geared mainly by the activity of the Na^+^/K^+^ pump known also as the Na^+^/K^+^ ATPase. We showed in a previous work [1] that epinephrine, a stress hormone, reduces the activity of the ATPase by binding to α2 adrenergic receptors and activating Src, p38MAPK, and ERK, leading eventually to PGE2 release. Although the literature reports an involvement of PGE2 in the regulation of ion and water transport across the colon [2], information regarding its effect on the pump is still scarce. This work is a follow up on the previous one and aims at determining the various signaling intermediates through which PGE2 inhibits colonic Na^+^/K^+^ ATPase using Caco-2 cells as a model. Unraveling the signaling pathway would have important clinical implications and would allow for the circumvention of any undesirable effect of PGE2, stress, and inflammation on colonic transport processes and water elimination.

## Materials and methods

### Materials

Dulbecco’s Minimal Essential Medium (DMEM) with 4500mg glucose/L and pyridoxine HCl, Fetal Bovine Serum(FBS), Trypsin-EDTA, Penicillin/Streptomycin(PS), 10x Phosphate Buffered Saline (PBS) without calcium and magnesium and (-)-Epinephrine were all obtained from Sigma,Chemical Co,St. Louis Missouri, USA.

Phorbol-12-myristate-13-acetate (PMA) and Calphostin C were purchased from Calbiochem, San Diago, USA. Glyco-SNAP1, Carboxy-PTIO, SC 19220 and NF-κB inhibitor were purchased from Santa Cruz Biotechnology, CA, USA, while PF-0441848, GW 627368, 8-Bromo-cGMP, and the PKG inhibitor KT 5823 were obtained from Tocris Bioscience, Bristol, UK. The inhibitor of soluble guanylate cyclase, ODQ, and Anti-alpha 1 Sodium Potassium ATPase (phospho Y10) antibody were purchased from Abcam MA, USA. Anti-IκBα mouse monoclonal antibody was obtained from R&D Systems, Minneapolis, MN, USA and anti-Na+/K+ ATPase α-1 Antibody was purchased from Merck, MA, USA.

The human colon carcinoma cell line, CaCo-2, was bought from American Type Culture Collection (ATCC), VA, USA. GAPDH monoclonal antibody was purchased from Cell signaling, MA, USA. Protease inhibitors cocktail tablets were purchased from Boehringer Mannheim, Germany. Clarity ECL Substrate, nitrocellulose membranes and Bio-Rad protein assay reagent were obtained from Bio-Rad, California, USA.

L-Ascorbic Acid, ouabain, prostaglandin E2 (PGE2), indomethacin, were procured from Sigma,Chemical Co,St. Louis Missouri, USA as well as all other chemicals.

### Methods

#### Culture of CaCo-2 cells

CaCo-2 cells were used at passages 25–32. They were grown, at a density of 120,000 cells/ml, on 100mm culture dishes in DMEM containing 4500 mg/ L^-1^ Glucose, sodium pyruvate, 1% Penicillin (100 μg mL^-1^), streptomycin (100 μg mL^-1^), 10% FBS, in a humidified incubator (95% O2, 5% CO2) at 37°C.

#### Treatment of CaCo-2 cells

At 90–100% confluence, cells were treated for 20min with either 0.5mM epinephrine dissolved in 0.5M ascorbic acid or with 1nM PGE2. They were then washed twice with PBS, lysed in presence of protease inhibitors, then scraped and homogenized at 4°C in a polytron at 22,000 rpm. Proteins were quantified colorimetrically at a wavelength of 595nm using the Bradford assay. The homogenates were then assayed for Na^+^/K^+^ ATPase activity as described below.

#### Membrane isolation

The cell homogenate obtained as described above was centrifuged at 720xg for 5min at 4°C. The supernatant was then collected and spun for 1 hour at 4°C in an ultracentrifuge at 100,000xg for membrane isolation. Proteins levels were determined in both membrane (pellet) and cytosolic (supernatant) fractions by the Bradford method. Eight microgram protein from every fraction were then loaded and resolved on 10% SDS polyacrylamide gel for western blot analysis as described below.

#### Na^+^/K^+^-ATPase activity assay

The activity of the Na^+^/K^+^ ATPase was assayed as described by Esmann [3]. Protein concentration of the homogenate was adjusted to 0.5μg/μl using a histidine buffer (150mM, pH 7.4), and samples were drawn and incubated for 30min at room temperature with 1% saponin, added at a ratio of 1:4(v/v,) in presence of a phosphatase inhibitor cocktail consisting of 10.7 mM glycerophosphate and 10.7mM pyrophosphate. Aliquots were then withdrawn and incubated at 37°C for an additional 30 min in histidine buffer containing NaCl (121.5mM), KCl (19.6 mM,), MgCl2 (3.92 mM), adenosine tri-phosphate (2.94 mM), in presence or absence of ouabain (1.47 mM), a specific inhibitor of the ATPase. The reaction was stopped by addition of 50% trichloroacetic acid at a ratio of 1:10 (v/v) and the samples were spun at 3000g for 5 min. The amount of inorganic phosphate liberated in the supernatant was measured colorimetrically at 750 nm according to the method of Taussky and Shorr [4]. Results are reported as μmol of inorganic phosphate liberated per μg protein per min and represented as a percentage of the control values which ranged between 0.074 and 0.041μmol/μg protein/min

#### The signaling pathway

**Type of prostaglandin receptors involved:** The activity of the Na^+^/K^+^ ATPase was assayed in Caco-2 cells treated with epinephrine (0.5 mM) or PGE2 (1nM) in presence and absence of specific antagonists to each of the EP receptors, namely SC 19220 (100 μM, DMSO; EP1 antagonist), PF-0441848 (1μM, DMSO; EP2 antagonist), L-798106 (10μM, DMSO; EP3 antagonist), and GW 627368X (10 μM, DMSO; EP4 antagonist). The inhibitors were added 20 min before the hormone or the prostaglandin.

**Involvement of PKC:** The involvement of PKC was investigated by pre-treating the cells for 20 min, with calphostin C (50nM, DMSO), an inhibitor of PKC. For further confirmation of the role of PKC, the cells were treated with Phorbol 12-myristate 13-acetate (PMA) (100nM, DMSO;20min), an activator of the kinase to see if it would imitate the effect of epinephrine/ PGE2.

**Involvement of nitric oxide:** The role of NO as a mediator was examined by studying the effect of epinephrine and PGE2 in presence of the nitric oxide scavenger Carboxy-PTIO (30μM; added 20min ahead). The effect of Glyco-SNAP1 (2μM; 20min), a nitric oxide donor was also investigated.

Since nitric oxide synthesis is under the control of NF-KB, the involvement of this transcription factor was investigated by studying the effect of epinephrine (0.5 mM) and PGE2(1nM) on the Na^+^/K^+^ ATPase in cells pre-treated for 30 min with an NF-KB inhibitor (15 μM, DMSO). The involvement of NF-κB was further confirmed by studying, by western blot analysis, the effect of PMA on the protein expression of IκB.

**Involvement of guanylate cyclase, cGMP and PKG:**One target of nitric oxide is the soluble guanylate cyclase (sGC) which generates cGMP. The involvement of the cyclase was tested by pre-incubating the cells for 20 min with ODQ (3μM, DMSO), a specific inhibitor of the enzyme, while the involvement of cGMP was investigated by incubating the cells with 8-bromo cGMP (0.5 mM, DMSO), a cell permeable cGMP analogue. cGMP activates PKG and the involvement of the latter was examined by investigating the effect of the PKG inhibitor KT5823 (1 μM, DMSO) on the ATPase in cells treated with the NO donor SNAP (2μM,20min).

**Locating the different mediators in the pathway:** Locating the mediators with respect to each other was determined via a similar procedure as the previous treatments.

The location of PKC with respect to PGE2 was investigated by pre-treating the cells for 20 min, with the COX inhibitor indomethacin, prior to the addition of PMA (100 nM,20min), or with calphostin C (50 nM) prior to the addition of PGE2 (1nM,20min).

The position of NO relative to PKC was determined by pretreating the cells for 20min, with PTIO (30 μM) prior to the addition of PMA (100 nM, 20min) or by pre-treating the cells for 20min with calphostin C (50 nM) prior to SNAP (2 μM, 20 min).

The location of NF-κB relative to PKC was examined by investigating the effect of an NF-κB inhibitor on the epinephrine/PGE2 induced decrease in the ATPase activity and by investigating by western blot analysis, changes in the protein expression of IκB. The position of PKG with respect to NO was determined by studying the effect of the NO donor SNAP, in presence of KT5823, an inhibitor of PKG.

### Western blot analysis

Equal amounts of proteins (40 μg) were loaded and resolved on 10% SDS polyacrylamide gel and transferred to a nitrocellulose membrane which was then blocked and incubated with an IκB antibody, followed by an incubation with a goat anti-rabbit secondary horse raddish peroxidase (HRP) conjugated IgG. The signal was detected by chemiluminescence using Clarity ECL Substrate. The intensity of the signal was detected using a ChemiDoc imager. GAPDH expression was used to check for equal loading. The bands were normalized to GAPDH using Image lab software.

### Statistical analysis

The results are reported as means ± SEM and tested for statistical significance by a one-way Analysis of Variance (ANOVA) followed by Tukey-Kramer multiple comparisons test using Instat and Excel Softwares.

## Results

### Epinephrine and PGE2 activate EP1 receptors

In a previous work epinephrine was shown to act on the Na^+^/K^+^ ATPase in Caco-2 cells via PGE2, and the effect of the hormone disappeared when all PGE2 receptors were blocked simultaneously [1].

**Fig 1:**
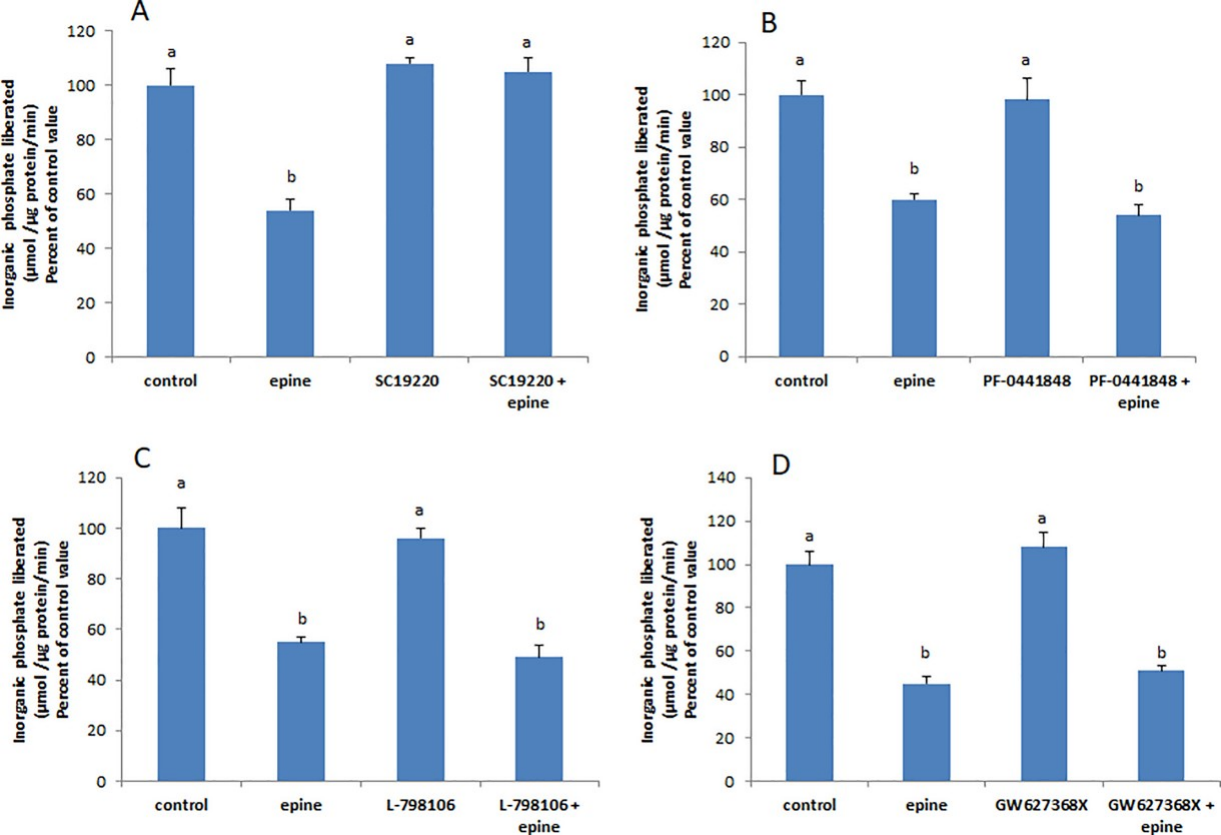
Epinephrine acts via EP1 receptors. The inhibitory effect of epinephrine (0.5mM, 20 min) disappeared in presence of (**A)** SC 19220, an EP1 antagonist (100 μM), but still appeared in presence of antagonists to **(B)** EP2(PF-7981106; 1μM), **(C)** EP3 (L-798106; 1μM) and (**D)** EP4 (GW 627368X; 10 μM). Values are means ± SEM of at least 3 observations. Bars not sharing a common letter are considered significantly different from each other at P<0.01.

**Fig 2:**
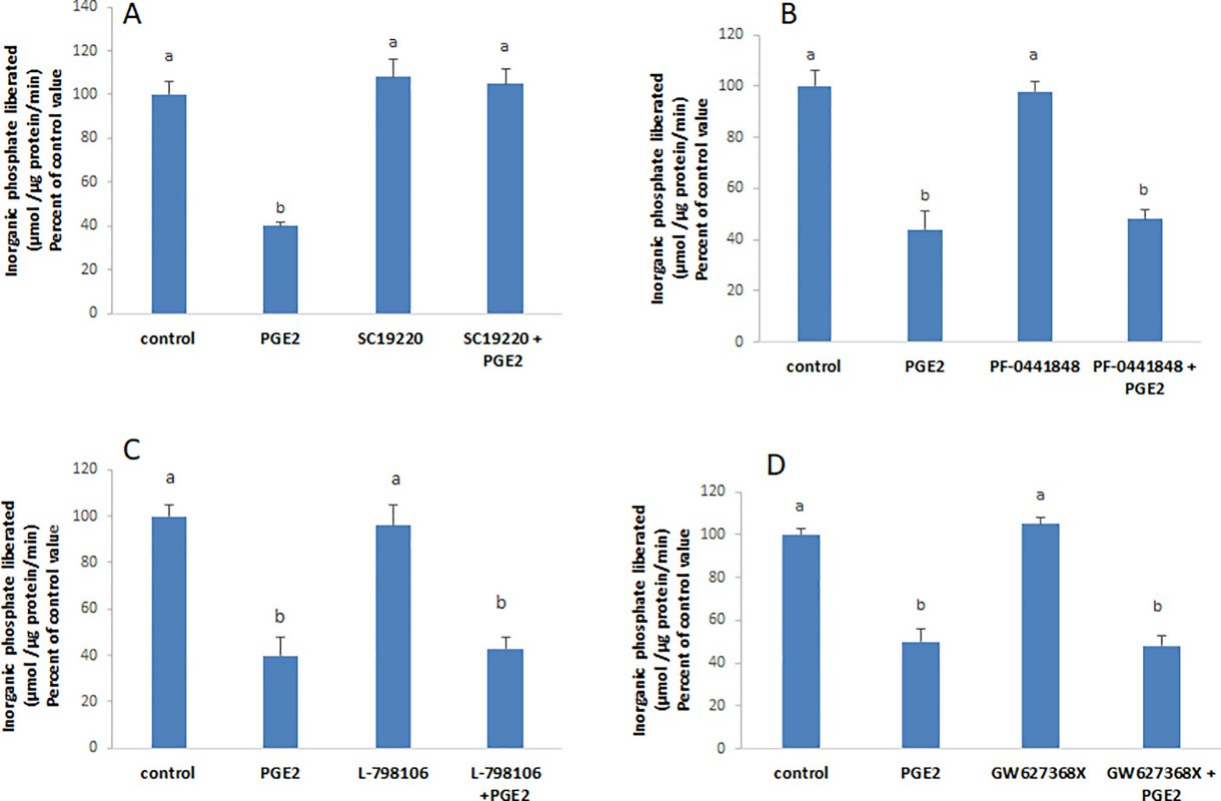
PGE2 binds to EP1 receptors. PGE2 (1 nM, 20 min) could not inhibit the Na^+^/K^+^ ATPase in presence of an antagonist to **(A)** EP1 receptors (SC 19220; 100 μM), while its effect appeared unaltered in presence of blocker of (**B)** EP2 (PF-7981106; 1μM), (**C)** EP3 (L-798106; 1μM) and **(D)** EP4 receptors (GW 627368X; 10 μM). Values are means ± SEM of at least 3 observations. Bars not sharing a common letter are considered significantly different from each other at P<0.01.

An attempt here was made to determine the receptor(s) involved, by treating the cells with epinephrine or PGE2 in presence of individual blockers of each of the receptors. The effect of the hormone and prostaglandin disappeared completely only when EP1 receptors were blocked with SC19220, and still appeared unaltered in presence of all other antagonists (Figs 1 and 2), suggesting an involvement of EP1 receptors only.

### Epinephrine and PGE2 activate PKC

**Fig 3:**
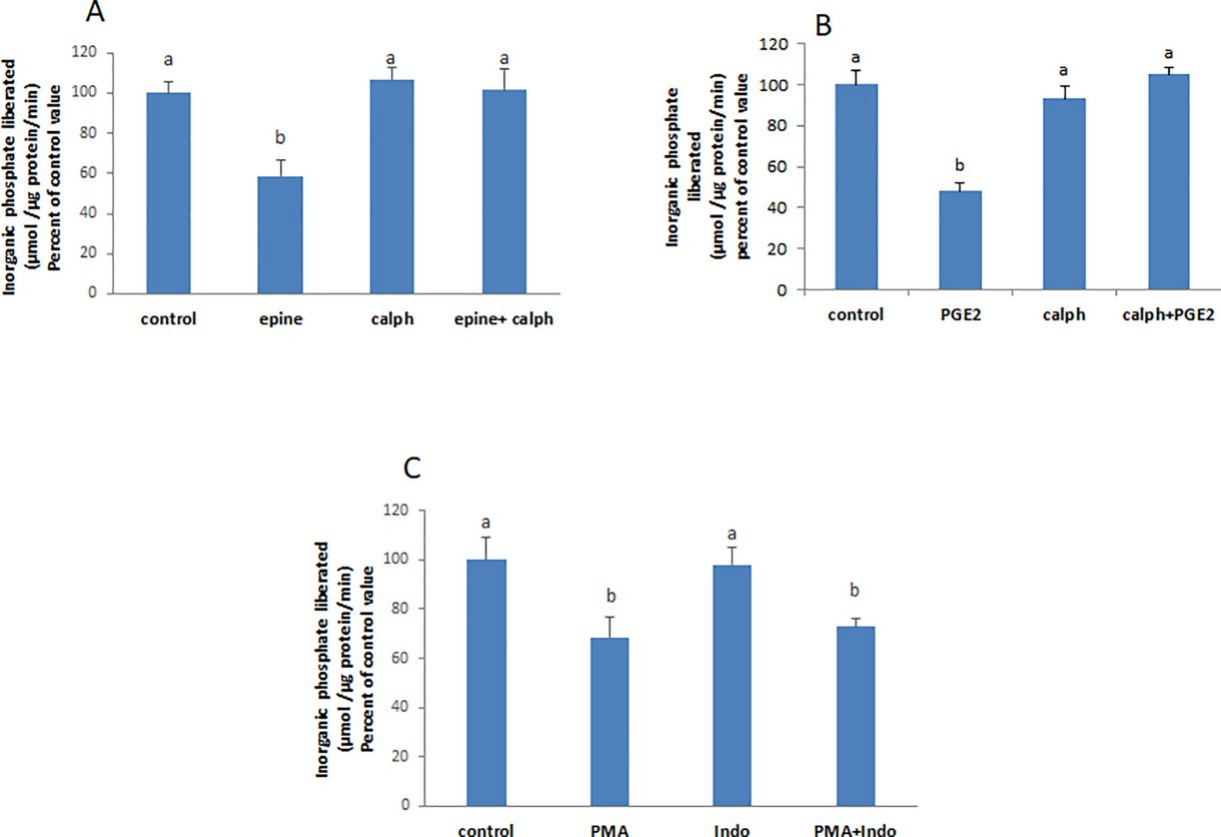
Epinephrine and PGE2 activate PKC. The effect of the (**A)** hormone and the **(B)** prostaglandin disappeared in presence of calphostin (50 nM), an inhibitor of PKC while **(C)** PMA, an activator of the kinase, exerted a significant inhibitory effect on the ATPase that was maintained in presence of indomethacin, an inhibitor of COX enzymes. Values are means ± SEM of at least 3 observations. Bars not sharing a common letter are considered significantly different from each other at P<0.01.

Because EP1 was found to be the receptor activated by epinephrine/PGE2, the involvement of PKC was suspected and investigated. The effect of epinephrine and PGE2 on the ATPase was abrogated in presence of calphostin C, an inhibitor of PKC (Fig 3A and 3B). PMA, an activator of PKC, imitated the effect of epinephrine and PGE2 and exerted a significant inhibitory effect on the ATPase. This effect was still manifested in presence of indomethacin (Fig 3C), an inhibitor of COX enzymes inferring that PKC is downstream PGE2.

### Epinephrine and PGE2 induce the release of nitric oxide

**Fig 4:**
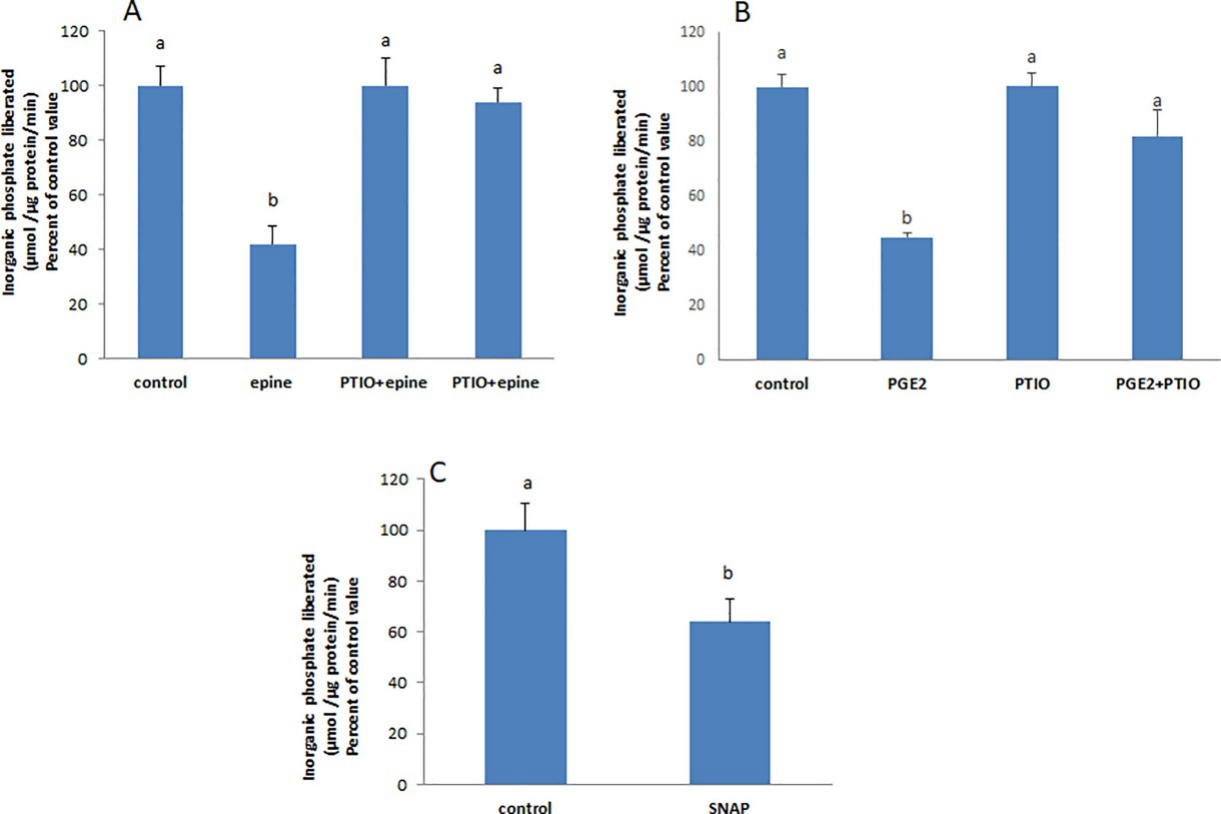
Epinephrine and PGE2 induce NO production. The effect of **(A)** epinephrine and **(B)** PGE2 did not appear in cells pre-treated for 20min with PTIO (30μM), a scavenger of NO. **(C)** SNAP (2μM, 20min) a NO donor caused a significant inhibition of the ATPase. Values are means ± SEM of at least 3 observations. Bars not sharing a common letter are considered significantly different from each other at P<0.01.

In many previous works, we showed that PGE2 modulates the activity of the Na^+^/K^+^ ATPase via nitric oxide (NO) [5,6]. In this work also, epinephrine and PGE2 did not exert their effect in presence of PTIO (Fig 4A and 4B), a scavenger of NO. In addition, the nitric oxide donor SNAP caused a significant inhibition of the Na^+^/K^+^ ATPase (Fig 4C).

### Epinephrine and PGE2 activate PKC which in turn activates the transcription factor NF-κB inducing NO production

**Fig 5:**
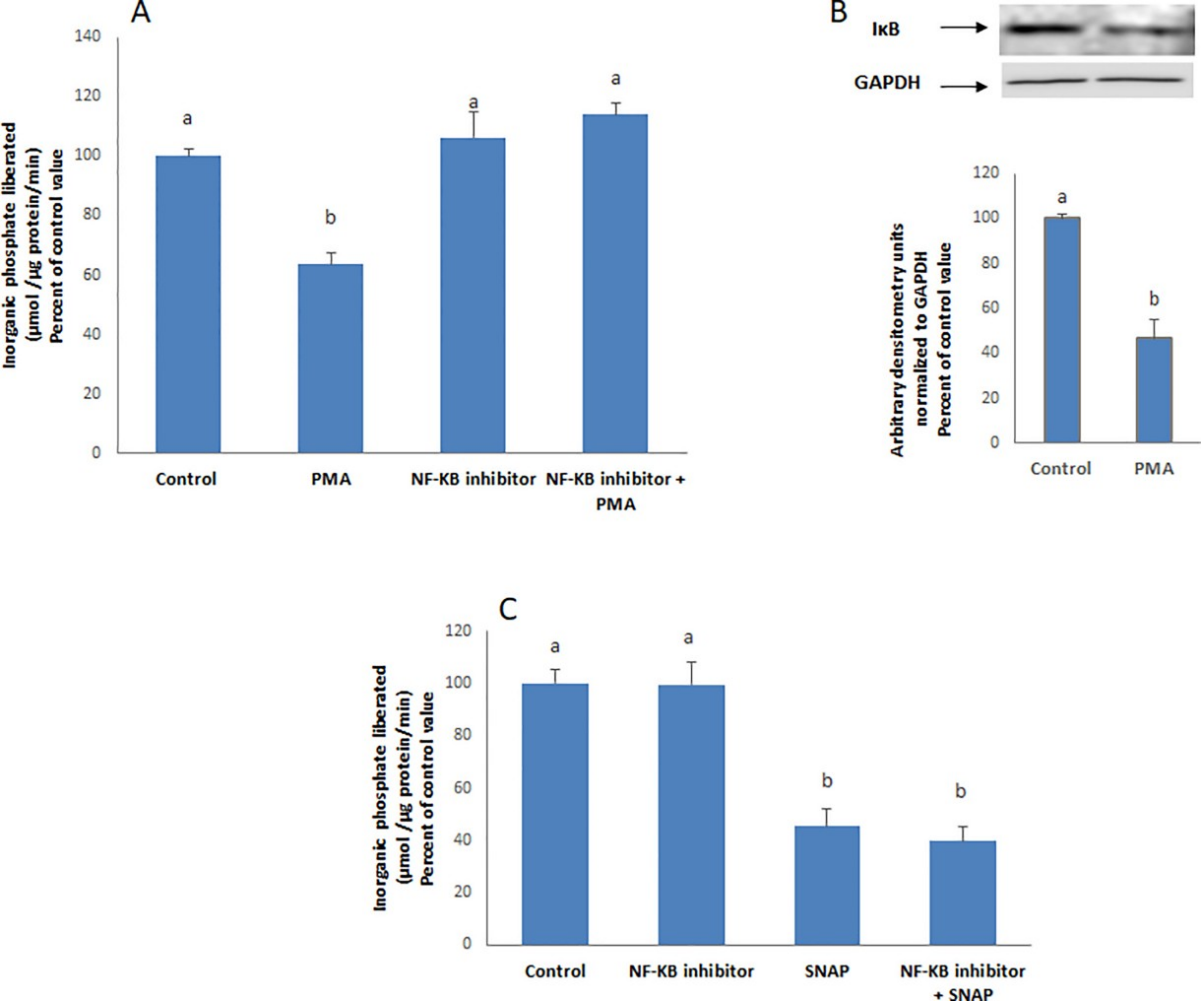
PKC activates NF-κB. **(A)** PMA (100 nM) could not inhibit the ATPase in cells pre-treated with an inhibitor of NF-**κ**B (15μM, 30 min) and **(B)** decreased significantly the protein expression of I**κ**B. The effect of (**C)** SNAP (2μM, 20 min) was not affected by the inhibitor. Values are means ± SEM of at least 3 observations. Bars not sharing a common letter are considered significantly different from each other at P<0.01.

Nitric oxide synthesis is catalyzed by the enzyme nitric oxide synthase (NOS), the iNOS isoform being the one induced by inflammation and various stimuli. PMA was reported to increase the activity of iNOS [7] which is under the control of the transcription factor NF-κB [8]. Inhibition of the transcription factor NF-κB abrogated the inhibitory effect of PMA (Fig 5A). The latter decreased the protein expression of IκB (Fig 5B) indicating that the transcription factor is downstream PKC.

The NF-κB inhibitor did not affect however the effect of SNAP on the ATPase (Fig 5C).

### Epinephrine and PGE2 activate the soluble guanylate cyclase and induce cGMP production and PKG activation

**Fig 6:**
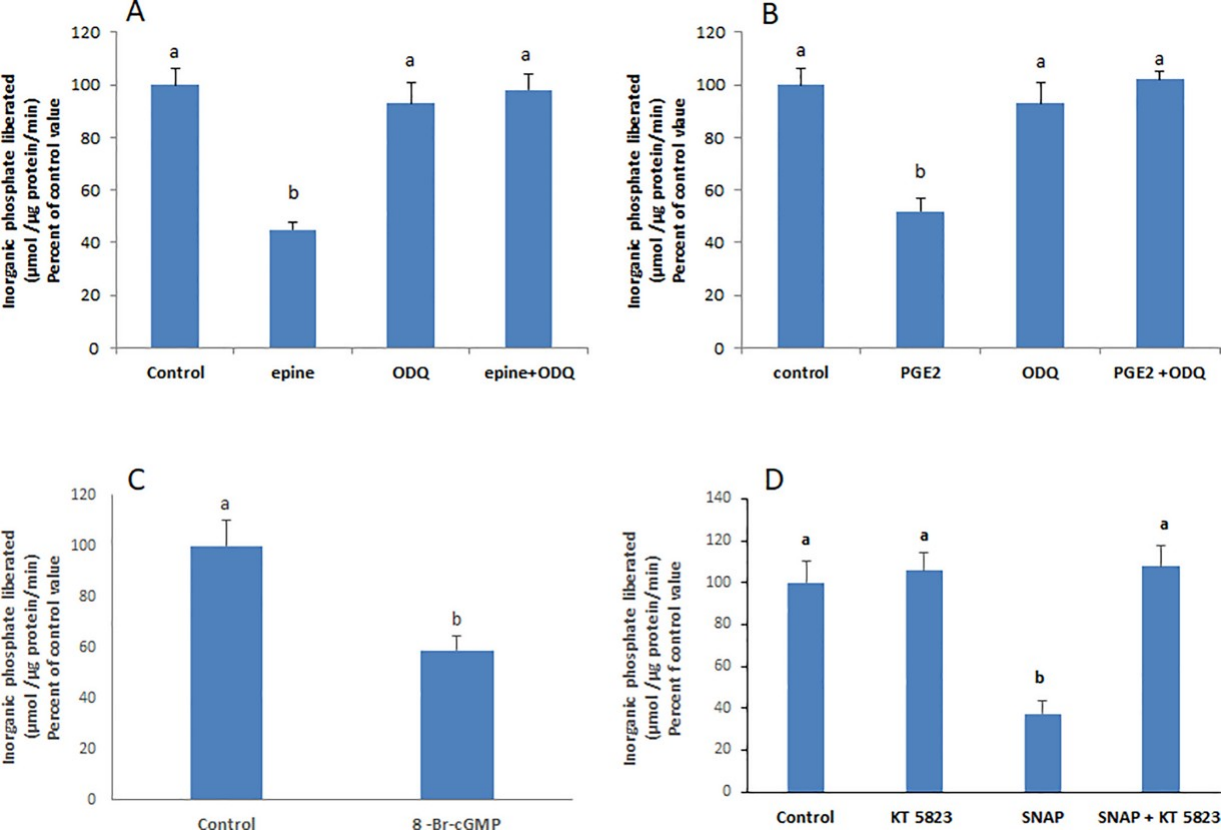
Epinephrine and PGE2 activate guanylate cyclase leading to cGMP production and PKG activation. Pre-treating the cells with ODQ (3μM, 20 min) abolished the effect of **(A)** epinephrine and **(B)** PGE2. **(C)** 8-Br-cGMP (0.5 mM, 20 min) exerted a significant inhibitory effect on the Na+/K+ ATPase. **(D)** SNAP (2μM, 20 min) did not inhibit the ATPase when cells were pre-incubate with KT 5823 (1 μM) an inhibitor of PKG. Values are means ± SEM of at least 3 observations. Bars not sharing a common letter are considered significantly different from each other at P<0.01.

The effect of epinephrine and PGE2 was not manifested when the soluble guanylate cyclase and PKG were inhibited respectively with ODQ and KT5823 (Fig 6A and 6B). 8-Br-cGMP, a cell permeable cGMP analogue, caused a significant inhibition of the Na^+^/K^+^ ATPase (Fig 6C).

### PKG is downstream NO

The effect of SNAP did not appear when PKG was inhibited with KT 5823 indicating that PKG is downstream NO (Fig 6D).

### PGE2 reduces the protein expression of the Na^+^/K^+^ ATPase

**Fig 7:**
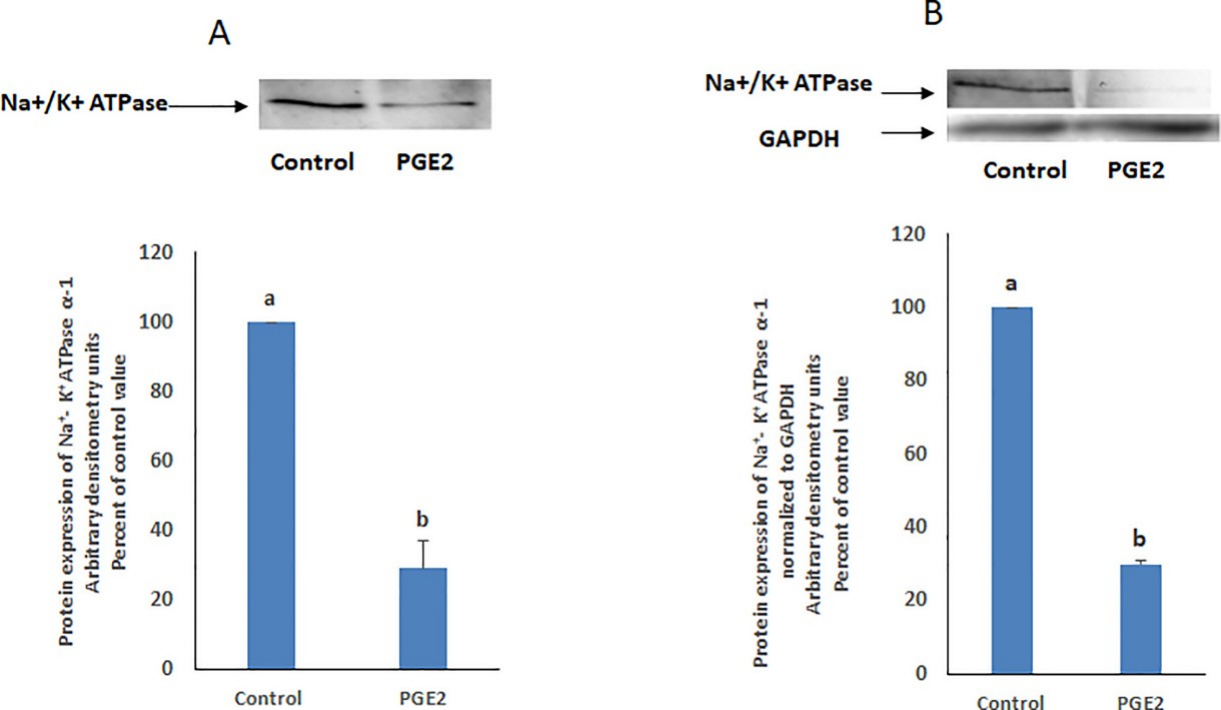
PGE2 reduces the protein expression of the Na^+^/K^+^ ATPase. Cells treated with PGE2(1 nM, 20 min) were collected and fractionated by differential centrifugation to isolate plasma membranes. Eight microgram samples from the cell homogenate **(A)** and from the membrane fraction **(B)** were used for western blot analysis. PGE2 reduced significantly the expression of the ATPase as indicated by the student t test (P<0.01). The blots are representative of an experiment repeated three times.

PGE2 reduced significantly the protein expression of the Na^+^/K^+^ ATPase in both the cell homogenate and the membrane fraction (Fig 7A & 7B), while the ATPase was not detected in the cytosolic fraction (Fig 7). No phosphorylated form at Tyr 10 was detected in any of the fractions or in the homogenate.

## Discussion

We demonstrated in a previous work that epinephrine reduces the activity of the Na^+^/K^+^ ATPase by inducing the release of PGE2 [1]. The data obtained here support the hypothesis that this change in activity results from a change in the abundance of the ATPase in the cell membrane. PGE2 acts via four subtypes of G-protein coupled receptors (EP1-4) [9]. Incubation of the cells with SC 19220 (selective blocker of EP1) prior to the treatment with epinephrine, completely abolished the inhibitory effect of the hormone (Fig 1), inferring that PGE2 acts by binding to EP1 receptors. Stimulation of the Gq-coupled EP1 receptors, activates PLC–dependent PIP2 hydrolysis increasing intracellular Ca2+ concentrations and PKC activity via IP3 and DAG respectively [10]. The observed activation of PKC by PGE2 supports further the involvement of EP1 receptors in the epinephrine/PGE2 induced inhibition of the Na+/K+ ATPase (Fig 1).

A similar inhibitory effect of PGE2 on the Na^+^/K^+^ ATPase was observed in another work [11] in which PGE2 release was induced by FTY720P. The prostaglandin there reduced the activity of the ATPase by activating however EP3 and not EP1 receptors. The involvement of both EP1 and EP3 in the modulation of the Na^+^/K^+^ ATPase activity has been reported before [12]. Because of different affinities, the type of receptor activated depends on the level of PGE2. EP3 has a very high affinity and is expected to be activated at low PGE2 levels, while the EP1 receptor, which has the lowest affinity among the 4 receptors, needs relatively higher levels [13]. Thus it seems that at the dose and time adopted in the two studies, epinephrine induced the release of higher levels of PGE2 than FTY720P.

Nitric oxide was found to be also a mediator in the pathway. NO synthesis is catalyzed by the enzyme nitric oxide synthase which exists in three isoforms, neuronal (nNOS), inducible (iNOS) and endothelial (eNOS). The inducible isoform is the one activated by inflammation and various stimuli, and the one involved in many stress pathologies [14,15,16]. It is known to be modulated by NF-κB [17,8,18] and is suspected to be here the one induced by epinephrine and leading to the release of NO. In the absence of any stimulus, NF-κB resides in the cytoplasm complexed with its inhibitory subunit IκB. Inactivation of IκB is achieved by phosphorylation by IκB kinase leading to its ubiquitination and destruction by the proteasome, relieving thus the transcription factor from its inhibitory effect, and allowing NF-κB to translocate to the nucleus where it modulates the expression of target genes [19]. In this work, a decrease in the protein expression of IκB was observed when cells were treated with PMA, indicating an activation of NF-κB that may be a direct or indirect result of phosphorylation by PKC. It could be concluded that activation of NF-κB by epinephrine/PGE2 was behind the production of NO and inhibition of the ATPase.

Two modes of NO-related regulation of the Na^+^/K^+^ ATPase have been described: cGMP-dependent and cGMP-independent.

The cGMP-independent mode is not very common. By interacting with other endogenously produced anions, such as superoxide, NO may generate free radical compounds that cause lipid oxidation and disruption of protein functions mainly by nitrosylating or nitrating critical amino acid residues [20, 21]. Guzman et al. [22] reported a role for peroxynitrite in the NO-induced inhibition of Na^+^/K^+^-ATPase in mouse proximal tubule epithelial cells. Moreover, nitrosylation of the Na^+^/K^+^-ATPase cysteine residues and thiol groups significantly reduced its activity in NO-treated brain and kidney tissues [23]. Other modification may include phosphorylation by NO-activated PKC as observed in OK cells that exhibited a PKG-independent but PKC-dependent decrease in Na^+^/K^+^-pump activity upon incubation with the NO generator sodium nitroprusside [24].

Soluble guanylyl cyclase (sGC) is a common and well recognized receptor for NO [25]. The binding of NO to the heme group of sGC induces the synthesis of cGMP, a potent activator of PKG [26]. The involvement of cGMP in the signaling pathway was demonstrated in this work by the observed inhibitory effect of 8-Br-cGMP, a cell permeable analogue of cGMP. The literature reports a cGMP/PKG induced decrease in the activity of the Na^+^/K^+^-ATPase in various cell lines including, mouse proximal tubule epithelial cells [22], opossum kidney cells [24], renal medulla [27], and non-pigmented ciliary epithelium of porcine eye [28]. Whether PKG alters the activity of the Na^+^/K^+^-ATPase directly via α-subunit phosphorylation or indirectly by the phosphorylation of other mediators needs still to be investigated.

Structural analysis revealed that PKA and PKG share common phosphorylation consensus sequences in substrate proteins [29]. Since PKA is known to directly target the Na^+^/K^+^-ATPase [30], PKG, therefore, should be capable of interacting directly with and phosphorylating the Na^+^/K^+^-ATPase; nonetheless, to our knowledge, literature supporting such a function is scarce and only few works reported phosphorylation of unidentified residues by PKG of the α- 1 subunit of Na^+^/K^+^-ATPase purified from the dog, sheep, pig, rat kidney, and Xenopus Oocyte [31]. This phosphorylation, however, resulted in a stimulation rather than an inhibition of the Na^+^/K^+^-ATPase.

PKG-indirect regulation of the Na^+^/K^+^-ATPase, on the other hand, is better understood and was shown to involve a variety of signaling mediators. cGMP/PKG elicited the phosphorylation and activation of DARPP-32 and protein phosphatase inhibitor -1(I-1) to inhibit protein phosphatase-1 and reduce the activity of the Na^+^/K^+^-ATPase in tubular [32] and kidney cells [33]. Furthermore, NO-dependent decrease in aqueous humor secretion in porcine eye was shown to be a consequence of cGMP/PKG- mediated ERK1/2 and p38-MAPK activation leading to phosphorylation of the Na^+^/K^+^-ATPase at Tyr-10 [34] and its inhibition. In this work however, no phosphorylated form at Tyr-10 was detected. Several unidentified intermediates may still the present between PKG and the Na^+^/K^+^ ATPase leading to a reduced activity through reduced abundance in the membrane. Another possible role of PKG would be the indirect phosphorylation of the Na^+^/K^+^ ATPase by a downstream kinase which may constitute the signal that induces internalization of the ATPase into endosomes via clathrin coated pits [35] and its eventual degradation.

Another reported cGMP-dependent modulation of the Na^+^/K^+^-ATPase is achieved via regulation of phosphodiesterases such PDE2 and PDE3.cGMP was shown to stimulate PDE 2 and inhibit PDE3 causing down-regulation and up-regulation of intracellular cAMP respectively [27]. Similarly, NO was reported to inhibit Na^+^/K^+^-ATPase in leptin-treated renal cells by the sequential stimulation of cGMP, activation of PDE2, and decrease in cAMP concentrations [36]. An increase in cAMP was also reported to negatively affect the Na^+^/K^+^-ATPase, in rat renal cortex [37] and mice hippocampus neurons [38], but via a mechanism independent of PDE3; nonetheless, the possibility that PDE3 can be responsible for such effects in other tissues can’t be ruled out.

In conclusion, our work demonstrated an inhibitory effect of epinephrine on the Na^+^/K^+^-ATPase in CaCo-2 cells that was mediated through PGE2. The prostaglandin acts via EP1 receptors and stimulates PKC which activates NF- κB and enhances iNOS expression and NO synthesis. The latter activates soluble guanylyl cyclase leading to cGMP production and PKG stimulation which inhibits the ATPase by phosphorylating an intermediate signaling molecules leading to a decrease in its expression in the membrane and consequently in its total activity.

The colon constitutes the major site for water and electrolyte absorption [39]. The Na^+^/K^+^-ATPase maintains a low intracellular Na^+^ concentration in colonocytes and provides the driving force for the passive entry of Na^+^ from the lumen [40]. A decrease in the activity of the Na^+^/K^+^-ATPase may result in a reduced colonic water absorption and may be behind the diarrhea that sometimes accompanies stress.

**Fig 8:**
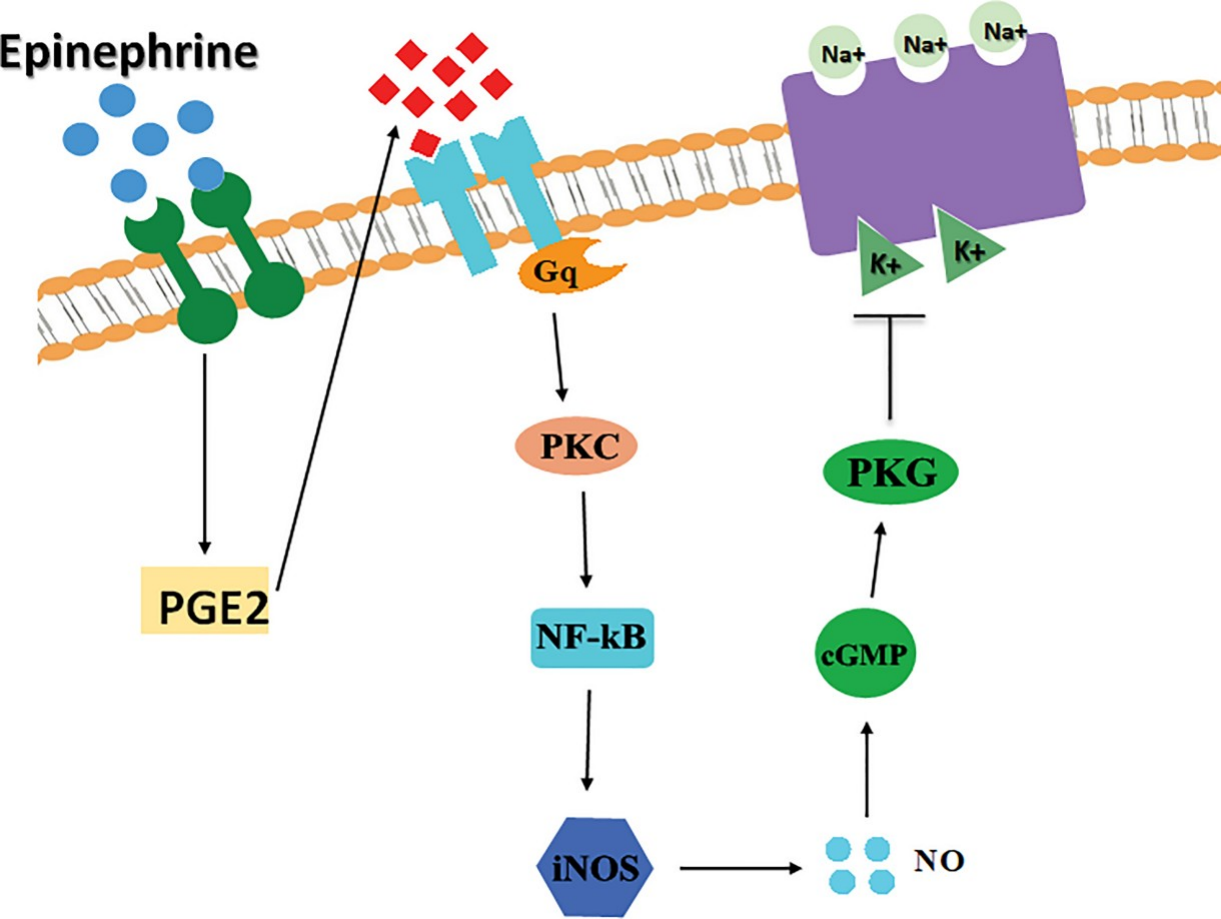
Proposed signaling pathway.

Fig 8 summarizes the signaling pathway mediating the inhibitory effect of epinephrine on the Na^+^/K^+^-ATPase
